# Photothermal Evaluation of Aqueous Magnetite Nanodispersions: Accuracy, Precision, and Limitations

**DOI:** 10.3390/molecules30204084

**Published:** 2025-10-14

**Authors:** Vladislav R. Khabibullin, Daria-Maria V. Ratova, Ksenia O. Andreeva, Yulia S. Vershinina, Ivan V. Mikheev, Sergei N. Shtykov, Mikhail A. Proskurnin

**Affiliations:** 1 Analytical Chemistry Division, Chemistry Department, M. V. Lomonosov Moscow State University, Moscow 119234, Russia; vladhab1995@gmail.com (V.R.K.); darmarrat@gmail.com (D.-M.V.R.); yu.vrshn@gmail.com (Y.S.V.); 2 Department of Analytical Chemistry and Chemical Ecology, Institute of Chemistry, Saratov State University, Saratov 410012, Russia; kazimirova-ks@mail.ru (K.O.A.); shtykovsn@mail.ru (S.N.S.); 3 Federal State Budgetary Institution of Science Institute of African Studies, Russian Academy of Sciences, Moscow 123001, Russia

**Keywords:** thermal lens spectrometry, nanoparticle dispersions, magnetite nanodispersions, surface functionalization, thermal diffusivity, steady state photothermal measurements, transient photothermal measurements

## Abstract

The thermal and optical properties of aqueous dispersions of magnetite nanoparticles were studied by dual-beam thermal-lens spectrometry. Surface-modified magnetite nanoparticles with an average crystal size of 7.5 nm were synthesized by a simple, one-stage method of coprecipitation followed by surface functionalization. For this purpose, the most popular and promising modifiers based on surfactants, polyelectrolytes, biopolymers and organic acids were used. The effect of the concentration of nanoparticles (in the range from 0.01 to 5 mg/L) and the nature of the surface modifier on the thermal diffusivity of the dispersion was studied. It was found that at concentrations of 0.4–0.6 mg/L, the dispersions exhibit heat-accumulating properties, which may be promising in the development of a magnetically controlled heat-conducting liquid. Thermal lens spectrometry in the steady-state measurement mode was used to reveal the processes of deposition and adsorption of magnetite nanoparticles on the surface of a quartz cell, leading to an apparent increase in thermal diffusivity by more than 30%. The paper touches upon the issues of accuracy and precision of temperature diffusion measurements, processing, and presentation of measurement results of time-resolved transient and steady-state signals for dispersed systems. The ratio of the change in the steady-state thermal-lens signals to the change in concentration regarding the concentration (d*ϑ*/d*c* vs. *c*) provides a way to identify a systematic error at a low level (less than 5%) of thermal-lens measurements caused by a high concentration (or optical absorption) of the object. Various options for signal normalization (in terms of power, absorbance, and pure-solvent signal) are considered, and their advantages and disadvantages are discussed. An approach to using thermal diffusivity as a function of the steady-state signal of the sample is proposed. This approach allows for a comparative thermal-lens analysis of objects with different optical and thermal properties.

## 1. Introduction

Photothermal spectroscopy (PTS) is a group of methods based on the registration of nonradiative relaxation of excited molecules [1,2,3]. A special place among the PTS methods is occupied by thermal lens spectrometry (TLS), which is based on the registration of changes in the refractive index of an object under the influence of external radiation. A high-power laser beam is used as a radiation source, which is absorbed when passing through the sample. The absorbed energy is dissipated as heat due to nonradiative relaxation, creating a thermal field that acts as an optical element similar to a lens (or thermal lens). The radiation beam passing through the thermal lens is expanded. This phenomenon underlies the registration of the analytical signal as a change in the size of center intensity of the probe beam [1,4].

Thermal-lens spectrometry has a higher detection sensitivity compared to transmission optical absorption spectroscopy techniques. Thus, TLS is used in colorimetric analysis [2,5,6,7,8,9,10], as a highly sensitive detection method in chromatography [11,12,13,14,15,16,17,18], and for the estimation of fluorescence or photochemical quantum yields [19,20,21,22]. The development of mathematical models has opened up the possibility of measuring thermal properties using thermal-lens techniques: thermal diffusivity, thermal conductivity, temperature coefficient of refractive index, and recording temperature changes [3]. Simultaneous recording of two signals (of thermal and optical nature) allows using TLS in studying the kinetics of chemical and photochemical reactions [23,24,25], and as a quality control method in the production of ultrapure solvents and fuels [26,27,28,29]. Successful use of thermal-lens spectrometry in biochemical studies [6,30], in the analysis of proteins [31], amino acids [32,33], blood components [7,34], and tissue studies [35,36] is reported. Thermolysis registration in microfluidic systems and in individual cells is gaining acceptance [37,38,39,40,41,42,43].

Currently, one of the main directions of TLS is the studies of dispersed systems, where heat-conducting nanofluids are being actively developed and there are tasks to analyze their thermophysical properties [44,45,46,47]. For such systems, thermal diffusivity, which is the rate of heat propagation when the temperature changes over time, plays a key role [48]. Here, TLS demonstrates higher sensitivity compared to traditional thermophysical methods. It is important that the method can detect differences in the morphological properties of nano- and microparticles [49,50,51], estimate the particle shell and its thickness, and identify physicochemical changes in dispersed systems (aggregation, sedimentation, chemical degradation, etc.) [28,52,53].

Among finely dispersed systems and new nanomaterials such as fullerenes, nanotubes, and nanodiamonds, magnetite (FeO,·Fe_2_O_3_, or Fe_3_O_4_) nanoparticles (NP) play a special role in applied chemical and biochemical studies [54,55,56]. This is due to its magnetic nature, ease of obtaining, biocompatibility, and low toxicity [56,57,58,59]. Easy surface functionalization (with silicon oxide, surfactants, polyelectrolytes, biopolymers, and organic acids [60,61,62,63]) of magnetite NPs increases their aggregation and sedimentation stability in liquids [64]. A wide variety of possibilities for magnetite functionalization provides a wide range of applications for nanoparticles: from magnetic solid-phase extraction to targeted drug delivery and magnetic hyperthermia [65,66]. The magnetic nature of magnetite opens up prospects for obtaining a new class of controlled magnetic heat-conducting liquids.

Even though magnetite is very widespread in applied studies, researchers have not fully investigated its thermal properties and the possibility of creating heat-conducting liquids from it using photothermal methods. Most times, thermal lens studies have focused on colloidal solutions of other nanomaterials like gold and silver NPs [67,68,69,70], as well as copper and zinc oxides [71,72,73,74], which are more stable in aqueous media than magnetite.

Against the background of the growing interest in the application of TLS in nanotechnology, difficulties arise associated with mathematical modeling and interpretation of the measurement results for such systems [68]. To date, there are several models for TLS that are successfully used to measure thermal and optical properties for homogeneous true solutions or transparent solid materials [75,76,77]. In such models, all accompanying effects (thermophoresis, convective mass transfer, concentration diffusion, photobleaching, etc.) of the main photothermal processes are usually excluded [75,77]. On the contrary, in complex multicomponent and dispersed systems, under the influence of radiation, thermal and optical effects arise that hinder the precise assessment of thermal properties [78,79,80,81,82]. Resolving these issues is difficult due to the lack of reference samples; thus, only generic approaches to sample preparation for photothermal analysis of complex systems can be used. A wide variety of finely dispersed multicomponent objects requires an individual approach in each individual case. In many sources, similar results of photothermal measurements are traced for various dispersed systems, which, on the one hand, may be associated with common mechanisms of heat transfer in heterogeneous systems, and on the other hand, common mathematical and technical features of recording and interpreting the results of photothermal measurements. Currently, there are two ways to process the results of thermal-lens measurements of dispersed systems:

(1) development of a specific mathematical model that considers all the thermal and optical effects and
(2) the use of models for homogeneous systems with additional restrictions and boundary conditions.

In the former case, the currently proposed improvements have limitations in applicability and have a cumbersome mathematical apparatus, complicating finding thermal and optical properties, which often require measurements of additional parameters by other methods [83,84,85]. Thus, the latter way—a model for homogeneous systems with additional restrictions—is much more common. Such approaches, with minimal mathematical changes in the common (homogeneous) thermal-lens models, provide the basis for measurements of both thermal and optical properties, but in a narrow range of absorbances and concentrations [53]. However, the issue of the correctness of the measurement results obtained using such simplified models of thermal lensing remains not fully resolved. Thus, it is necessary to develop general algorithms for comparing results of photothermal measurements, which would allow photothermal experiments to be carried out under conditions of interlaboratory reproducibility.

The aim of this study is the measurement of thermal and optical properties of dispersed systems of surface-modified magnetite nanoparticles using dual-beam thermal lens spectrometry. The influence of the surface modifier of magnetite on the thermal diffusivity is studied. Particular attention is paid to the form of presentation of the results of steady-state and transient signals, general patterns and features of various mathematical normalizations. A comparative analysis of dispersed solutions with high and low optical absorption is carried out and recommendations for interpreting photothermal results for a wide range of heterogeneous systems are proposed.

## 2. Results and Discussion

The primary attention in this study is paid to the results of photothermal measurements of steady-state and time-resolved transient signals for aqueous dispersions of magnetite NPs. Particular attention is also paid to the mathematical normalization of signals and methods of presenting the results. Finally, the original mathematical processing of photothermal results is tested on systems of various natures: dispersions of graphene oxide, polystyrene nanoparticles, and silicon dioxide. The results of magnetite nanoparticle characterization, surface modification, and parameterization of aqueous dispersions are presented in the Supplementary Materials.

The most common magnetite surface modifiers were used in the work: citric acid (CA), sodium dodecyl sulfate (DDS), sodium polystyrene sulfonate (PSS), chitosan (CS), and cetyltrimethylammonium bromide (CTAB). Coating nanoparticles with citric acid (Fe_3_O_4_/CA) is a widespread method of particle stabilization [86,87,88,89]. Negatively charged hydrophilic carboxyl groups of citric acid stabilize the hydrophobic core well in water and prevent aggregation and sedimentation of particles [86,87]. Aqueous dispersions of magnetite nanoparticles are well stabilized by polyelectrolytes (Fe_3_O_4_/PSS), surfactants (Fe_3_O_4_/DDS, Fe_3_O_4_/CTAB) and polymers (Fe_3_O_4_/CS) due to functional groups and this approach is often used in biomedical applications [90,91], in targeted drug delivery and magnetic hyperthermia and tomography [54]. The choice of synthesis and modification methods is based on their simplicity and ease of implementation.

### 2.1. Steady-State Measurements

Steady-state signal (*ϑ*), largely dependent on light absorbance and concentration of chromophore particles. With an increase in the number of light-absorbing particles (nanoparticles or molecules), the steady-state signal increases. As mentioned previously, the relationship between the steady-state thermal-lens signal and the particle concentration is successfully used in quantitative colorimetric analysis [8,92].

**Figure 1:**
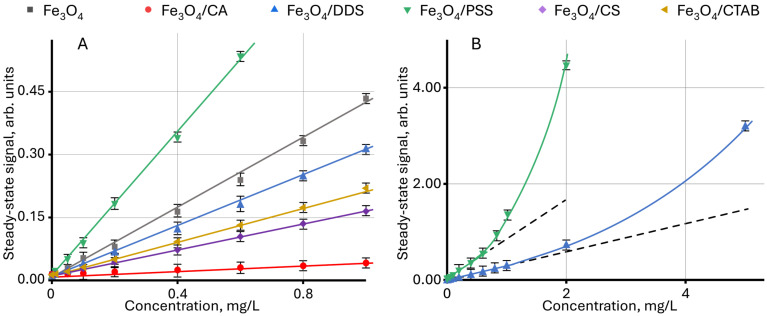
Effect of the concentration of modified magnetite nanoparticles in an aqueous dispersion on the steady-state signal (Equation (7)): (**A**) for a concentration of up to 1 mg/L; (**B**) for Fe_3_O_4_/PSS and Fe_3_O_4_/DDS samples (*n* = 3).

Figure 1 shows the dependence of the steady-state signal on the concentration for nanoparticles coated with various modifiers. For all dispersions, an increase in the steady-state signal occurs with an increase in NP content. It is noted that for nanoparticles modified with PSS, the rate of signal increase is the highest, and for Fe_3_O_4_/CA, the lowest. The different slope angles of the linear dependences show different contribution of the surface modifier to the steady-state signal, which can be used in problems of identifying nanoparticles by the modifier type. It is interesting to note that for some samples, a change in the linear dependence is observed at high concentrations (Figure 1B).

**Figure 2:**
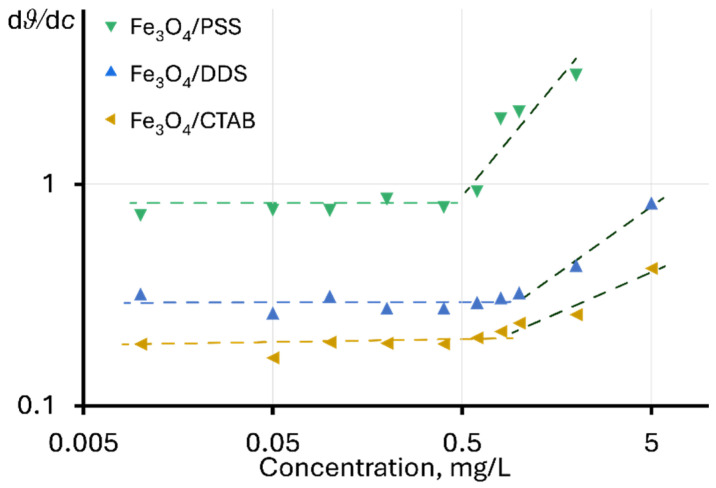
Dependence of the ratio of the change in the steady-state signal to the change in concentration on the concentration of nanoparticles in the dispersion.

In the explicit form, similar changes are observed for Fe_3_O_4_ dispersions modified with PSS, DDS, and CTAB, when using the dependence of the ratio of the change in the steady-state signal to the change in concentration on the concentration d*ϑ*/d*c* vs. *c*, as shown in Figure 2. In this case, the value of d*ϑ*/d*c* for the linear section of the function *ϑ* = f(*c*) is constant, and the change in d*ϑ*/d*c* indicates a violation of linearity. In this case, the highest deviation is observed for Fe_3_O_4_/PSS, and the lowest for Fe_3_O_4_/CTAB. For unmodified NPs and particles modified with chitosan and citric acid, no violations of linearity are observed in the concentration ranges used.

A change in d*ϑ*/d*c* with concentration may indicate a change in the heat transfer mechanism in dispersions, affecting the development of a temperature gradient under the action of excitation radiation. It is important to note that negative deviations from linearity are usually observed at high values of light absorption, which prevents the passage of radiation into the cell (the phenomenon of thermal lens “saturation”) [93]. Positive deviations may be observed for surfactant solutions or at high electrolyte contents [80,94,95], due to their effect on the thermooptical properties of the solvent (mainly d*n*⁄d*T*) [96]. In [80], positive deviations from linearity for a steady-state signal were noted with an increase in molecular weight of the surfactant. In our case, the greatest signal enhancement is observed for PSS, whose molecular weight is significantly higher than that of DDS and CTAB (approx. 70,000 versus 288 and 364 g/mol, respectively).

**Figure 3:**
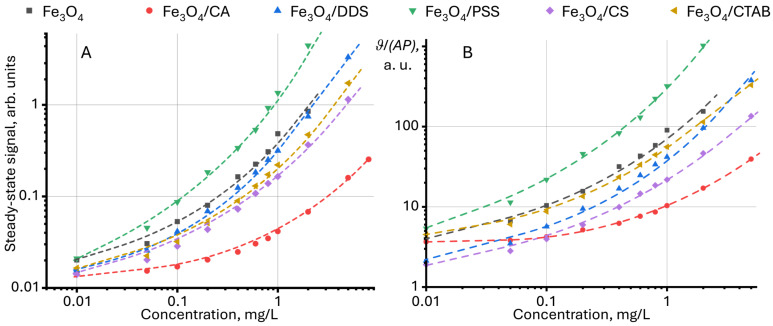
Dependence (**A**) of the steady-state signal (Equation (7)) and (**B**) relative to the absorbance and power of the steady-state signal on the concentration of modified magnetite nanoparticles in an aqueous dispersion (*n* = 3).

In the existing data, the steady-state signal normalized to the power of the excitation beam and/or the absorbance (*ϑ*/*P* or *ϑ*/*P*/*A*) of the test sample is used to present the results of steady-state signal measurements [97,98,99,100,101]. This approach considers excitation power and absorbance, which allows for unified results to be compared between different objects. Figure 3 shows the dependence of the steady-state signal and the relative absorbance and power of the steady-state signal on the concentration. As seen from the normalization results, the shape of the curves is like each other.

In this study, we propose an additional normalization of the relative steady-state signal to the value for the solvent (in our case, for water), found by the following equation:

(1) $$Relative\;stationary\;signal=\frac{{\vartheta /(PA)}_{sample}}{{\vartheta /(PA)}_{solvent}}$$

Representation of the steady-state signal in this form allows us to additionally exclude the effect of thermooptical properties of the solvent on the sample signal.

**Figure 4:**
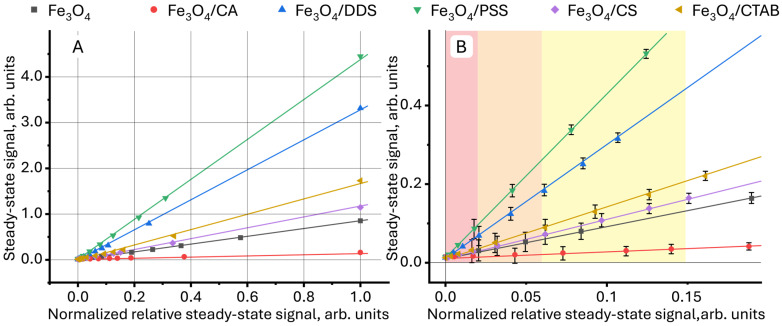
Dependence (**A**) of the steady-state signal (Equation (7)) on the relative steady-state signal (Equation (1)), normalized in the range 1÷0 for aqueous dispersions of magnetite nanoparticles with different surface modifiers, in which the green rectangle is the initial section presented in detail in (**B**).

Further normalization of the relative steady-state signal between 1÷0 and representation of the steady-state signal as a function of the normalized relative steady-state signal ϑ = *f*(*normalized relative steady-state signal*) provides linear dependences that can be used in quantitative analysis (Figure 4). Deviations from linearity (which are observed in Figure 1) are not observed. The different slopes of the straight lines (Figure 4A) indicate a different contribution of the surface modifier to the value of the steady-state signal for each sample, which can be used to identify NPs.

Different sensitivities of quantitative determination of nanoparticles with different surface modifiers in aqueous media divides the linear concentration range for samples into three regions, schematically shown in different colors in Figure 4. In the red region, the lowest concentrations, it is impossible to distinguish all the images from each other due to the high RSD (7–10%). In the middle, orange region, it is possible to distinguish aqueous dispersions of Fe_3_O_4_/PSS and Fe_3_O_4_/DDS both from each other and from other samples. The relative standard deviation in this region was 5–7%. In the yellow region, it is possible to distinguish magnetite modified with citric acid from uncoated NPs and particles modified with CTAB and chitosan, showing the lowest change in the slope. In this region, the relative standard deviation of the steady-state signal measurement is 3–5%. Above the normalized relative steady-state signal of 0.15, all samples have significant differences, and the RSD does not exceed 2%.

#### Long-Term Steady-State Measurements

Continuous long-term thermal-lens measurements of the steady-state and transient signals make it possible to study physicochemical processes [1,2,3]. For dispersed systems, this opens up the possibility of studying morphological changes and assessing the degradation of components. Previously, two processes were found to occur simultaneously for an aqueous dispersion of graphene oxide (GO): decomposition and partial chemical reduction of GO [52].

**Figure 5:**
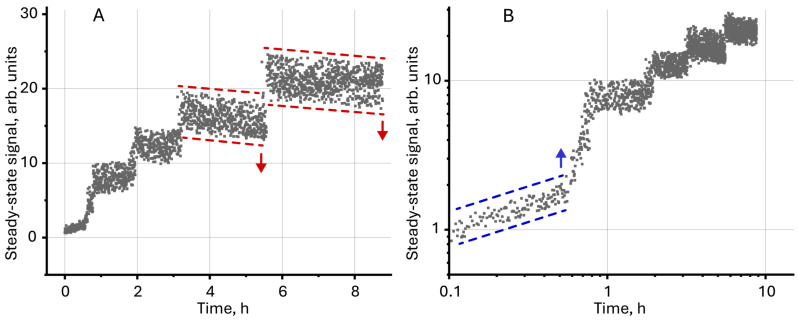
Steady-state signal (Equation (7)) for unmodified magnetite (*c* = 2 mg/L) measured over several hours of continuous thermal lens measurements in (**A**) normal and (**B**) logarithmic scale.

Unmodified magnetite nanoparticles are characterized by low colloidal stability and sedimentation resistance, which leads to aggregation and sedimentation of particles over time [59,102]. A decrease in the concentration of NPs due to their sedimentation reduces absorbance, which negatively affects the steady-state signal. In our case (Figure 5), on the contrary, an increase in the steady-state signal was detected over several hours of continuous photothermal measurements. The signal growth is accompanied by a sudden increase (Figure 5B). Such behavior for dispersed systems (as well as true solutions) has not been previously detected and, at first glance, contradicts the concepts described above. A possible justification for the observed effects may be the irreversible sorption of particles (or an ensemble of particles) on the walls of the cell in the laser transmission zone. Then, when the next particle is sorbed from the solution volume on the cell wall, the apparent absorbance of the solution increases and the steady-state signal grows.

The initial growth of the signal, which is observed during the first 30–40 min (Figure 5B), indicates the aggregation of individual particles in the volume. This leads to an increase in the actual particle size and absorbance. The observed drop in the signal after 3–4 h (Figure 5A) indicates the sedimentation of particles at the bottom, leading to a decrease in absorbance. At the same time, the observed phenomena (sedimentation and aggregation) do not have a noticeable effect on the overall dynamics of the steady-state signal, which confirms the proposed hypothesis about the sorption of particles on the cell walls.

Similar dynamics of the steady-state signal is characteristic of systems with a chemical reaction leading to an increase in absorbance due to a decrease in the concentration of the initial components with low absorbance and an increase in the content of reaction products with high absorbance [103]. For modified magnetite nanoparticles, no changes in the steady-state signal were observed during 8–10 h of measurements, which indicates stabilization of dispersion due to modification of the NP surface.

### 2.2. Time-Resolved Transient Thermal-Lens Measurements

Time-resolved transient thermal-lens measurements are necessary to find thermal properties (mainly thermal diffusivity and thermal conductivity), as well as to study secondary thermal and optical effects that arise under the influence of external radiation [1,2,3]. Important parameters for assessing the heat-conducting properties of nanofluids are viscosity *ρ*, thermal conductivity *k*, and heat capacity *C_p_* [104,105]. Thermal diffusivity *D* according to the well-known equation *D* = *k*/(*C_p_*∙*ρ*) combines these parameters and is a key characteristic value for assessing the properties of liquids including dispersed (nano)fluids. Using thermal diffusivity, it is possible to identify and assess the processes of chemical and photochemical degradation [28], as well as aggregation and sedimentation [53], which are important criteria for the stability of dispersed systems.

**Figure 6:**
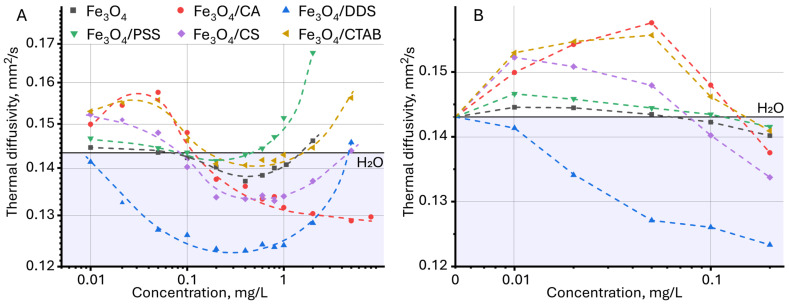
Dependence of thermal diffusivity on the concentration of nanoparticles, (**A**) over the entire range of concentrations studied and (**B**) the initial section, up to 0.2 mg/L (*n* = 3).

For dispersions of magnetite nanoparticles, the effect of NP concentration and modifier nature on thermal diffusivity was studied. First of all, it was noted that with increasing concentration for all the studied samples, a complex dynamic of thermal diffusivity change is observed, which can be divided into several stages (Figure 6A). At the first stage, in the concentration range up to 0.05 mg/L for all samples except Fe_3_O_4_/DDS, an increase in thermal diffusivity is observed, with highest for citric acid, CTAB, and chitosan. At the second stage, at a concentration from 0.05 to 0.4–0.6 mg/L, a significant decrease in thermal diffusivity occurs. At the third stage, at concentrations above 0.6–0.8 mg/L, the dynamics changes again and for all samples except Fe_3_O_4_/CA, and an increase in the thermal diffusivity of the dispersion is observed.

The variable behavior of thermal diffusivity may be associated with a change in the heat-transfer mechanism in solution. To understand the phenomena occurring, it is necessary to consider the main factors affecting thermal diffusivity. The thermal diffusivity of a dispersion is affected by many factors: the nature and morphology of nanoparticles, the presence and thickness of the shell, the concentration of NPs, pH, etc. [69]. According to the equation *D* = *k*/(*C_p_*∙*ρ*), an increase in thermal conductivity leads to an increase in thermal diffusivity, while an increase in heat capacity and/or density leads to its decrease. As a rule, the presence of a dispersed phase reduces the heat capacity of an object [74]. This is due to the fact that the specific heat capacity of heat-conducting nanofluids is always less than that of a solvent, since solids have a comparatively lower heat capacity, and this increases the thermal diffusivity of the system [74]. Nanofluids have a low concentration of the dispersed phase at the level of several mg/mL (< 1%), and the change in density can be neglected. Thus, thermal diffusivity depends largely on thermal conductivity, but it is also not necessary to state that this is the main factor influencing thermal diffusivity. Separately, several other important factors are distinguished for thermal diffusivity, including Brownian motion [74,106] and phonon–phonon scattering, i.e., energy transfer from the nanoparticle to the liquid [73,107]. Here, an increase in particle size and concentration leads to an increase in phonon scattering [68,108] and an increase in thermal diffusivity. It is reported [73,109] that smaller particles have poor contact with the solvent and phonon scattering transfers less energy to the surrounding liquid, which reduces thermal conductivity and, subsequently, thermal diffusivity. At a subtler level, it is assumed that liquid molecules form layered structures around solid surfaces of nanoparticles, and these monolayers play an important role in enhancing thermal diffusion [110].

Thermal diffusivity and thermal conductivity are also affected by particle morphology [111,112], presence of defects, ligation [113], and inclusions of other elements in the particle structure [114]. A study showed that thermal diffusivity for a nanofluid with carbon black annealed at 300 °C decreases by 20% compared to carbon black annealed at 400 °C, which the authors attribute to a change in the structural modification of carbon [107]. Thermal diffusivity of a nanofluid also increases with absorbance [74], nanoparticle size [49,69,73,115], and aggregation [116]. The authors [69] explain this effect based on the positive effect of particle size on the thermal conductivity of nanofluids. As the average particle size increases, the total solid/liquid interface area decreases, which leads to a weakening of the interfacial thermal resistance effect and an increase in the thermal conductivity of nanofluids [117]. Aggregation and clustering also reduce the specific heat of particles [71], which has a positive effect on thermal diffusivity. On the other hand, particle sedimentation reduces thermal diffusivity [73].

In the studied case, the initial increase in thermal diffusivity may be associated with both an increase in the Brownian motion of particles and a decrease in the heat capacity of dispersion. As the concentration of nanoparticles in the dispersion increases, the specific heat capacity decreases leading to an increase in thermal diffusivity, which was previously observed for aqueous dispersions of zinc oxide nanoparticles [110] and a zirconium-oxide–graphene-oxide composite [74].

**Figure 7:**
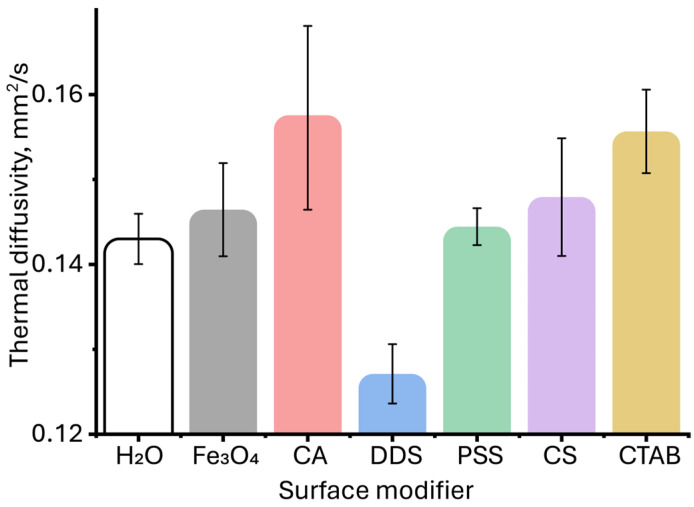
Thermal diffusivities of aqueous dispersions of magnetite nanoparticles with different surface modifiers at the same nanoparticle concentration of 0.05 mg/L. (*n* = 5).

A detailed examination of the region of the initial increase in thermal diffusivity revealed a change in the relative standard deviation (RSD) of the assessment of thermal diffusivity in comparison with pure water (Figure 7). At first glance, the value of the measured thermal diffusivity for unmodified magnetite particles and those coated with citric acid, PSS, chitosan, and CTAB demonstrates a relative standard deviation lying within the experimental error of measuring thermal diffusivity for water. This may indicate the absence of significant changes in the thermal properties for these particles at low concentrations. However, as established previously [53], in photothermal analysis of dispersed systems, the error value of the found thermal diffusivity can be an indicator of the presence of a dispersed phase, and the occurrence of various physicochemical processes (aggregation, sedimentation, etc.), and can be used as an additional signal to identify qualitative differences in the samples [118]. This was established for solvents, where differences in the steady-state signal, thermal diffusivity, and relative standard deviation of thermal diffusivity measurement were observed at the same absorbance and close viscosity and density [53]. Similar observations were made [83], where the authors found a slight increase in thermal diffusivity for aqueous solutions of carbon nanotubes, which was within the error limits for the solvent. Thus, despite the apparent lack of differences for some samples and pure water, the initial increase in thermal diffusivity in TLS is significant. In general, for all samples, with increasing concentration, the relative standard deviation of the thermal diffusivity measurement decreases: if in the 0.01–0.05 mg/L range, it is 5–10%, then by 1 mg/L it decreases to 1% (Figure S1, Supplementary Information).

The decrease in thermal diffusivity observed after 0.05 mg/L may be due to the decrease in Brownian motion due to the steric factor: according to the dynamic light scattering (DLS) technique, the average hydrodynamic size of coated particles exceeds several thousand nanometers. This leads to a hindered motion of the nanoparticles relative to each other, with increasing concentration. Clustering and aggregation increase the viscosity and reduces thermal diffusion; when the concentration of nanoparticles exceeds the optimal value, the dispersion stability decreases, and aggregation and/or agglomeration of nanoparticles decrease the thermal diffusivity [74,110]. As the size increases, Brownian motion also decreases, and the interaction between the particles and the liquid medium decreases, leading to lower thermal diffusivity values [119,120]. For smaller particles, intense Brownian motion leads to easy thermal diffusion, which results in faster heat dissipation [106], which is observed for magnetite particles coated with citric acid. As the particle size increases, the total surface area and the number of defect states also decrease, which reduces nonradiative recombination and thermal diffusivity [120].

In this concentration range (from 0.05 to 0.1 mg/L), the effect of a surface modifier on the thermal diffusivity of the dispersion is observed. However, it is not yet possible to identify any specific patterns. As previously established, the coating agent also plays a role in reducing thermal diffusivity [49]. In [120], it was found that coating the surface of quantum dots with 2-mercaptosuccinic acid slows down the cooling of nanoparticles due to a decrease in the rate of heat transfer to the solvent. The increase in thermal diffusivity in dispersions above 0.6 mg/L (Figure 6A) can be associated with an increase in heat radiation in the near field and heat transfer between two nanoparticles that are close to each other. Although aggregation can reduce Brownian motion as noted above due to the increased mass, heat diffusion can be increased due to the bond between the particles [121]. Each aggregate can be considered as a new particle with higher thermal conductivity and an effective radius equal to the radius of gyration. In addition, it was found that when the distance between nanoparticles is less than their characteristic sizes, thermal conductivity is two to three orders of magnitude higher than when two nanoparticles are in contact [122], which is a possible reason for the observed sharp increase in thermal diffusivity.

Thus, thermal properties of the dispersion can be controlled by changing the concentration of nanoparticles: at a concentration of up to 0.05 mg/L and above 2–5 mg/L, the dispersions mostly exhibit good heat-conducting properties, and in the range of 0.2–0.8 mg/L, heat-accumulating properties.

#### Long-Term Measurements

**Figure 8:**
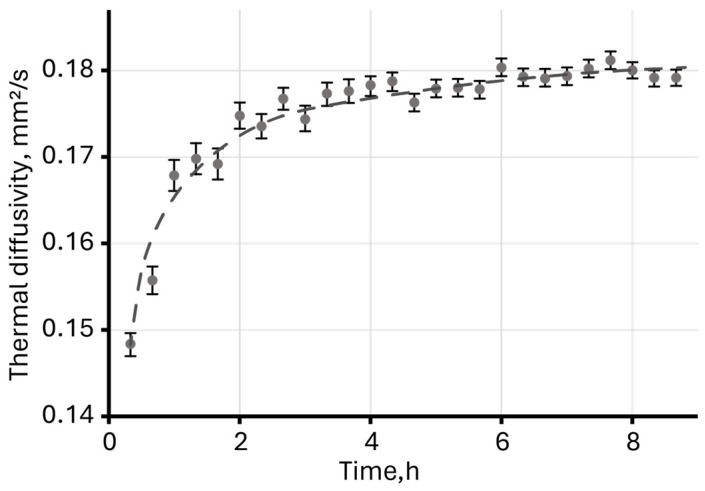
Thermal diffusivity of an aqueous dispersion of unmodified magnetite measured over eight hours (*c* = 2 mg/L).

Long-term photothermal measurements of an aqueous dispersion of magnetite nanoparticles revealed a sharp increase in thermal diffusivity (Figure 8) during the first 2–3 h, followed by reaching an equilibrium, constant value.

A change in the steady-state signal over 9 h showed possible sedimentation and adsorption processes on the walls of the cell in the laser-irradiation zone leading to a sudden increase in the signal and, at the same time, a slight decrease in the signal, clearly observed after 4 h of measurements (Figure 5A).

An increase in thermal diffusivity in dispersed systems, as well as in true solutions, may indicate the occurrence of physicochemical processes [53]. Changes in thermal diffusivity were observed for true solutions of substituted phthalocyanines [123,124], as well as aqueous dispersions of graphene oxide (GO) [52]. In the first case, thermal diffusivity was influenced by the strong solvation of molecules, which was subject to change due to the chemical degradation of substituted phthalocyanines over time, and in the case of graphene oxide dispersion, two processes were observed: decomposition and partial photoinduced chemical reduction of GO, which also led to an increase in thermal diffusivity.

Since magnetite nanoparticles in an aqueous medium without surface modification are unstable and aggregate and precipitate over time, a possible explanation for the observed increase in thermal diffusivity may be confirmation of the adsorption processes on the cell glass surface proposed above. This leads to an apparent increase in concentration and absorbance, which, as stated above, directly affects the thermal diffusivity of the solution [74].

### 2.3. A New Approach to the Treatment of Thermal-Lens Transients

In the analysis of the results of photothermal measurements of disperse systems, the literature suggests several approaches to processing steady-state and transient signals:

(1) selecting the characteristic time $t_{c}$ and thermooptical signal *θ* to approximate the model and experimental transient curves [68,110];
(2) using the concentration expressed as the number of particles per unit volume [125];
(3) normalizing to the power of the excitation beam and the absorbance of the sample of the steady-state signal [52];
(4) normalizing in the range 1–0 of transient curves on a logarithmic scale and using the initial section of the transient curve (100–500 ms) to find the thermal diffusivity [115].

The use of the proposed approaches ensures the accuracy of the results of measurements of thermal properties with an error of less than 5% but requires considering the optical and morphological features of the test samples and performing additional measurements (absorbance, particle size, etc.).

**Figure 9:**
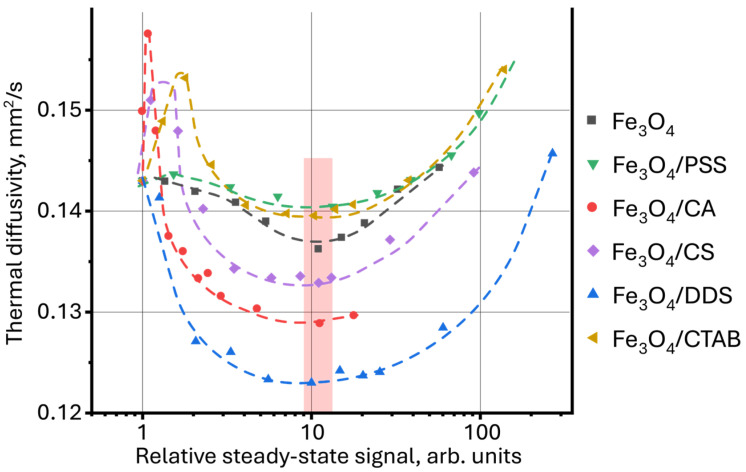
Dependence of thermal diffusivity on the relative steady-state thermal-lens signal (Equation (1)) for aqueous dispersions of magnetite nanoparticles with different surface modifiers.

In this study, it is proposed to use thermal diffusivity as a function of the relative steady-state signal, found by Equation (1). (Figure 9). In this case, the thermal diffusivity for all samples reaches a minimum value at a relatively steady-state signal of ca. 10. It is important to note that the dynamics of changes in thermal diffusivity for all samples become the same, which was not observed when using the dependence *D* = *f*(*c*) in Figure 6A. This may result from the influence of thermooptical properties of nanoparticle surface modifiers, which, despite the differences, have the same magnetite core. Here, the NP surface modifier contributes to the value of thermal diffusivity, and the nature of magnetite in the general dynamics of changes in the thermal properties of dispersions.

**Figure 10:**
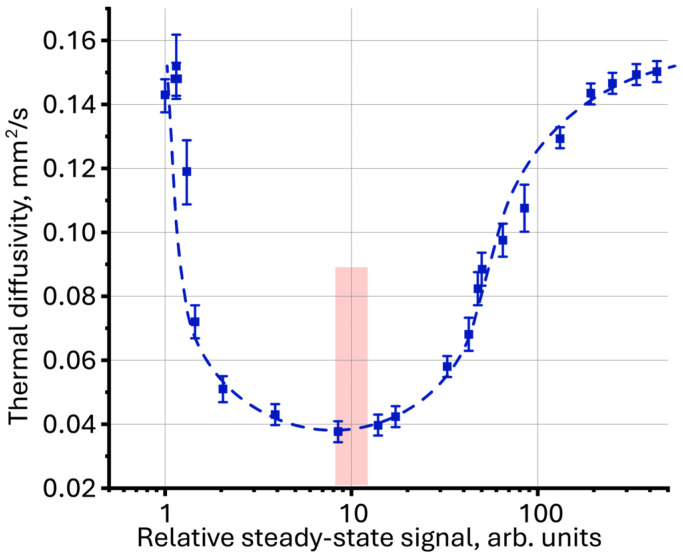
Thermal diffusivity as a function of relative steady-state signal (Equation (1)) for an aqueous dispersion of graphene oxide (fraction < 14 kDa, *n* = 3).

Using thermal diffusivity as a function of the relative steady-state signal, a comparative analysis of the photothermal measurement results for aqueous dispersions of graphene oxide, silicon oxide and polystyrene nanoparticles, measured previously [52,115,125], was performed. The proposed interpretation of the measurement results revealed new patterns previously not discovered for these systems. For graphene oxide dispersions, for which similar dynamics of thermal diffusivity change with increasing concentration is observed as for magnetite, the lowest value of thermal diffusivity was obtained with a relatively steady-state signal of ca. 10 (Figure 10), which also coincides with the results for magnetite nanoparticles described in this work.

**Figure 11:**
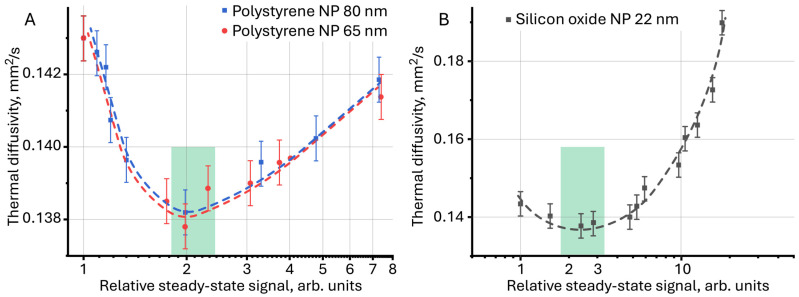
Thermal diffusivity as a function of relative steady-state signal for (**A**) aqueous dispersions of polystyrene nanoparticles and (**B**) for aqueous dispersions of silicon oxide (*n* = 5).

Comparison of the results of photothermal measurements for surface-painted and unpainted polystyrene particles (Figure 11A), obtained previously [125] using a new approach, made it possible to identify the general dynamics of the behavior of thermal properties both among themselves and between particles of different sizes, which was impossible to achieve in the classical representation as *D* = *f*(*c*). The lowest thermal diffusivity is achieved with a relative steady-state signal of ca. 2, which is also observed for aqueous dispersions of silicon oxide (Figure 11A,B, green rectangles).

This may be due to the thermooptical properties of the objects and the individual contribution of the particles to the thermal diffusivity of the dispersion. In addition, magnetite and graphene-oxide nanoparticles are objects with high light absorption, and dispersions of silicon oxide and polystyrene nanoparticles, on the contrary, are samples with low absorbance and rather high light scattering.

For a more accurate understanding of the found patterns, further research in this area is required. The discovered pattern may be the key to classifying nanomaterials according to their influence on thermal, thermo-optical and optical properties of dispersions.

Thus, the proposed approach using thermal diffusivity as a function of the relative steady-state signal allows for a comparative analysis of results of photothermal measurements of thermal diffusivity for samples of different natures without additional studies of particle morphology. The ratio of the steady-state signal to the excitation laser power, absorbance, and signal of a pure solvent allows for the thermooptical properties of the medium to be considered and the contribution of the surface-modified particle to the thermal lens signal to be revealed.

Further work can be aimed at more in-depth studies of the relationship between the steady-state thermal-lens signal and thermal diffusivity. It is important to test the proposed approach on a wide range of nanomaterials and complex multicomponent systems. It is also necessary to identify and study the influence of measurement parameters and optical scheme geometry on the thermal diffusivity function from the steady-state signal. Since the absorption of one laser photon and its conversion into thermal energy is the primary mechanism for excitation of a thermal lens, it is important to study the influence of excitation laser wavelength [83].

## 3. Materials and Methods

### 3.1. Chemicals

The following chemical reagents were used in the work:

- Iron(II) chloride tetrahydrate, (FeCl_2_·4H_2_O, 99.8%) “Acros Organics”, (Geel, Belgium);
- Iron(III) chloride hexahydrate, (FeCl_3_·6H_2_O, 99.8%) “Acros Organics”, (Geel, Belgium);
- Cetyltrimethylammonium bromide (CTAB, 98%) “Sigma-Aldrich”, (St. Louis, MO, USA);
- Sodium dodecyl sulfate (DDS, 95%) “Servicebio”, (Wuhan, China);
- Sodium poly(4-styrenesulfonate) (PSS, M_r_ ca. 70,000) “Sigma-Aldrich”, (St. Louis, MO, USA);
- Chitosan (CS, Mr ca. 50–190,000) “Sigma-Aldrich”, (St. Louis, MO, USA);
- Citric acid (C_6_H_8_O_7_, 99.5%) “Acros Organics”, (Geel, Belgium);
- Sodium hydroxide (NaOH, 99.8%) “Ecros” (St. Petersburg, Russia).

All solutions were prepared using ultrapure water obtained from a Milli-Q system, Millipore Corporation (Millipore SAS, Molsheim, France).

### 3.2. Preparation Aqueous Dispersions of Modified Magnetite Nanoparticles

Magnetite nanoparticles were synthesized by the method of coprecipitation of iron salts in an alkaline medium, described [126]. Surface modification was carried out in an aqueous medium by mixing an aqueous dispersion of magnetite NPs with a modifier solution of a certain concentration. The methodology for the synthesis and surface modification of magnetite nanoparticles is presented in the Supplementary Materials; the data are coincident with the previous data on this material [57,60,127].

The preparation of working dispersions was carried out by successive dilution of an aqueous dispersion with a concentration of 100 mg/L with deionized water.

### 3.3. Characterization of Nanoparticles

X-ray diffraction measurements (Figure S2, Table S1) were carried out on a Bruker D2 PHASER diffractometer (Bruker, Billerica, MA, USA) using Cu Kα radiation (*λ* = 0.154 nm) at 40 kV and 30 mA over a range of 2*θ* values (10° to 80°). The size and shape of the nanoparticles (Figure S3) were measured using a Tecnai-G2 F20 transmission electron microscope (FEI Company, Hillsboro, OR, USA) with a spatial resolution of up to 0.2 nm. The hydrodynamic size (z-average size) and zeta potential (*ζ*-potential) of the samples (Table S2) were recorded by dynamic light scattering (DLS) using a Nano-ZS Zetasizer, model ZEN3600 (Malvern Instruments Ltd., Malvern, UK) at an angle of 173°. The absorption and IR spectra (Figure S4) were recorded using a Shimadzu UV-2550 spectrophotometer (Shimadzu, Kyoto, Japan) and a Bruker 70 FTIR spectrometer (Bruker, Ettlingen, Germany). A commercial Grad 57–35 ultrasonic bath with a generator power of 165 W (Grad-Technology, Moscow, Russia) was used for dispersion preprocessing [128,129]. The results of the characterization of nanoparticles are presented in the Supplementary Materials.

### 3.4. Photothermal Measurements

**Figure 12:**
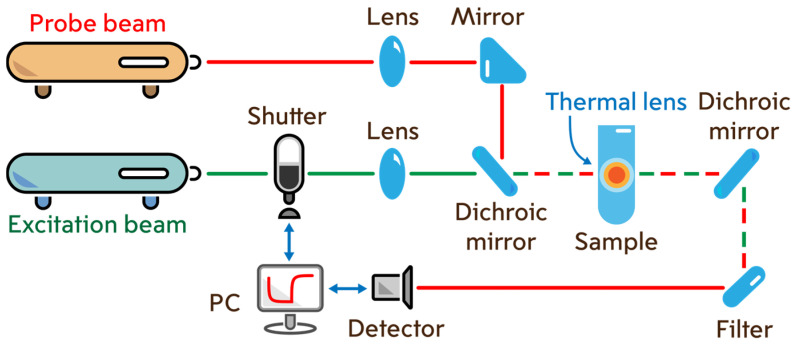
Dual-beam thermal lens spectrometer.

Steady-state and time-resolved transient photothermal measurements were carried out on a dual-beam thermal lens spectrometer in the mode-mismatch mode (Figure 12). The general principle of spectrometer operation and signal measurements is the continuous recording of periodic laser-induced heating of the sample. Two beams are used for this purpose: excitation and probe. The excitation beam is the radiation of a high-power (up to 500 mW) solid-state laser MGL-FN-532 (Optoelectronics Tech. Co., Ltd, Changchun, China) with a wavelength of 532.0 nm and a TEM_00_ mode composition. The probe beam is the radiation of a low-power (5.0 mW) helium-neon laser HNL050L (ThorLabs, Newton, NJ, USA) with a wavelength of 632.8 nm and a TEM_00_ mode composition. The excitation beam passes through an SH05 shutter (ThorLabs, Newton, NJ, USA), which is controlled by a c8051Fx-DK analog-to-digital converter board (Silicon Labs, Boston, MA, USA) connected to a personal computer (PC) and enters the sample cell. The radiation passes through the sample, partially absorbing and emitting as heat, which forms a density field that acts as an optical element similar to a lens (or thermal lens). After passing the cell, the excitation laser beam is cut off by a light filter. The probe beam, passing through the thermal lens in a coaxial (with the excitation beam) direction, expands. The beam expansion is recorded on a photodiode located in the far field as a change in intensity.

A single measurement cycle starts when the excitation beam shutter opens and continues after it closes. The cycle ends and starts again when the shutter opens again. Measurement cycles are generated, displayed and stored as transient curves (signal intensity versus time) [130]. The operating parameters of the measurements are given in Table S3.

The laser power was measured using Optronics Nova II (Ophir Optronics Solutions, Jerusalem, Israel).

### 3.5. Processing of Results of Steady-State and Transient Photothermal Measurements

The results of steady-state and time-resolved transient measurements were processed using the Shen and Snook model [131] with minor modifications. The basic equation for the change in intensity of the probing laser over time *I*(*t*) can be represented as follows:

(2) $$I\left(t\right)=I\left(0\right){\left[1-0.5\theta {tan}^{-1}\left(a/\left(b\left(t_{c}/2t\right)+c\right)\right)\right]}^{2},$$

where *I*(0) is the probe beam intensity at the initial moment of time *t* = 0, *θ* is the thermo-optical signal; *a*, *b*, *c* are the geometric constants of the thermal lens spectrometer, $t_{c}$ is the characteristic development time of the thermal lens.

The thermooptical signal *θ* depends on the thermal conductivity *k*, the temperature coefficient of the refractive index d*n*⁄d*T*, the linear absorption coefficient *α*, the optical path length *l*, the power of the excitation radiation *P*, the wavelength of the probing laser, respectively *λ*_*p*_ according to the following equation:

(3) $$\theta =\frac{P\alpha l}{k\lambda_{p}}\left(-\frac{dn}{dT}\right).$$

Geometric constants of the spectrometer *a*, *b* and *c* depend on the ratio of the beam waist sizes in the sample *m* and the geometric parameter *V* as follows: *a* = 2*mV*, *b* = (1 + 2*m*)^2^ + *V*^2^, *c* = 1 + 2*m* + *V*^2^. The beam ratio *m* and the geometric parameter *V* depend on the radii of the probe ($\omega_{p1}$) and excitation ($\omega_{e0}$) beams in the sample, the distance from the probe beam waist to the cell ($z_{1}$), the Rayleigh length for the probe beam ($z_{c}$) and the distance from the sample to the detector ($z_{2}$) through the following equations:

(4) $$m=({\omega_{p1}/\omega_{e0})}^{2},$$

and

(5) $$V=z_{1}/z_{c}+z_{c}/z_{2}\left[1+{\left(z_{1}/z_{c}\right)}^{2}\right].$$

The characteristic time of development of a thermal lens $t_{c}$ is related to the thermal diffusivity *D* according to the following equation:

(6) $$t_{c}=\omega_{e0}^{2}/4D.$$

Knowing the geometric parameters of the spectrometer, the intensity of the beams at each moment in time, the thermal conductivity and d*n*/d*T* (reference values), one can find the characteristic time and thermal diffusivity of the sample.

To find the steady-state signal (*ϑ*), the following equation was used [75]:

(7) $$\vartheta =\frac{I\left(0\right)-I\left(\infty \right)}{I\left(\infty \right)},$$

where $I\left(\infty \right)$ is the intensity of the probe beam at full development of the thermal lens (in a steady-state state).

The steady-state signal normalized to the absorbance and the power of the excitation laser is found using the following equation:

(8) $$\vartheta^{\prime }=\frac{\vartheta }{\alpha P}.$$

Since the Shen and Snook model is intended for processing the photothermal results of homogeneous and highly dilute solutions (where there are no side thermal and optical effects) [132], an adaptation was made for processing the results of dispersed systems, described in detail previously [75].

According to the proposed approach, from Equation (2), the characteristic time can be represented as a function of the development time of the transient curve according to the following equation:

(9) $$\tilde{t_{c}}\left(t\right)=[(a/tan[2\cdot (1-\sqrt{I\left(t\right)/I\left(\infty \right)})/\theta \prime ])-c]\cdot 2t/b$$

where $\tilde{t_{c}}\left(t\right)$ is the effective characteristic time at each moment of the transition curve development. In the case of a true solution or in the absence of thermophoresis, the steady state $I\left(\infty \right)$ is understood as the average value of the last points of the transient curve. In the case of thermophoresis, as well as for heterogeneous systems, a modified intensity of the probe beam $I\prime \left(\infty \right)$ was used, which is obtained by approximating the first 100–150 ms of the transient curve in such a way that the last points (of the 100–150 ms section) of the experimental curve fully correspond to the theoretical one. The transition from the effective $\tilde{t_{c}}$ to the true characteristic time $t_{c}$ occurs by averaging the values of the first 100–150 ms $\tilde{t_{c}}\left(t\right)$. Then, using Equation (6), the thermal diffusivity of the object is found.

Since the accuracy of photothermal measurement results depends on factors related to the mathematical model, spectrometer geometry and measurement parameters, it is necessary to check the correctness of photothermal measurements before analyzing target objects [52,115]. For this purpose, pure solvents with precisely known thermal and optical parameters are usually used [133]. In our case, distilled water and ethanol were used. The results of thermal diffusivity measurements for water and ethanol were 0.143 ± 0.003 and 0.090 ± 0.001 mm^2^/s, respectively, which is in good agreement with the literature data [53,67,134].

## 4. Conclusions

Approaches to interpreting thermal measurement results for multicomponent and disperse systems are of key importance, as they affect the accuracy of measurements of thermal and optical properties. A comparative analysis of photothermal results for various samples, as well as the results obtained on various photothermal spectrometers, is currently presented only through the dependence of thermal diffusivity and steady-state thermal-lens signal on the content or morphological features of the system components. The approach by which these results were obtained requires additional measurements, which complicates the work. The lack of common certified approaches and reference samples, which makes unification of results virtually impossible, aggravated this problem. The complexity of developing common approaches also lies in the photothermal spectroscopy method itself, where steady-state and transient signals nonlinearly depend on the morphological, optical, and thermal properties of test samples. In this study, thermal and optical properties of magnetite nanoparticle dispersions were investigated and approaches to processing the results of photothermal measurements were considered using graphene oxide, polystyrene, and silicon oxide dispersions as examples. Magnetite nanoparticles with an average size of 7.5 ± 2.5 nm were synthesized by the coprecipitation method in one stage, after which the surface of the nanoparticles was functionalized with modifiers of various nature. Successful surface modification was confirmed using IR spectroscopy and zeta potential measurements. By thermal lens spectrometry, the effect of the dispersed phase concentration in the range of 0.01–5 mg/L and the nature of the surface modifier on the thermal and optical properties of magnetite nanoparticles were studied. It was found that with an increase in concentration from 0.01 to 0.4–0.6 mg/L, the thermal diffusivity decreases to 0.120 mm^2^/s, and a further increase to 2–5 mg/L leads to an increase in thermal diffusivity that reaches 170 mm^2^/s. The use of a steady-state signal made it possible to identify differences between modifiers for particles, using a steady-state signal relative to the value of the pure solvent as an argument of the function. Thermal lens measurements carried out for 6 h for a dispersion of unmodified nano-sized magnetite revealed a complex dynamic of changes in the steady-state signal and an apparent increase in thermal diffusivity of more than 30%, indicating the occurrence of aggregation, sedimentation and adsorption of particles on the cell surface. To compare different dispersion samples, it was proposed to use thermal diffusivity as a function of the relative steady-state signal. This approach made it possible to recognize a similar effect of the nature of the object on the thermal properties for particles with high and low light absorption, which had not been discovered previously.

## Data Availability

The original contributions presented in the study are included in the article/Supplementary Materials, further inquiries can be directed to the corresponding author.

## Supplementary Materials

The following supporting information can be downloaded at: https://www.mdpi.com/article/10.3390/molecules30204084/s1, Figure S1: Relative standard deviation of thermal diffusivity measurements of magnetite nano-particle dispersions (*n* = 3); Figure S2: Diffraction pattern of uncoated and chitosan, PSS- and DDS-modified magnetite nano-particles; Figure S3: TEM images for the Fe_3_O_4_; Figure S4: ATR-FTIR spectra in the absorption mode from 4000 to 400 cm^−1^ (64 scans, 2 cm^−1^) of: (A) pure nanoparticles; (B) particles doped with CA; (C) particles doped with CS; (D) particles doped with CTAB; (E) particles doped with DDS; (F) particles doped with PSS; Table S1: Estimation of crystallite sizes by the Williamson–Hall method; Table S2: Average hydrodynamic size and zeta potential of magnetite nanoparticles with different surface modifiers; Table S3. Thermal-lens measurement parameters.

## Abbreviations

The following abbreviations are used in this manuscript:

NP	Nanoparticle
TLS	Thermal-lens spectrometry
PTS	Photothermal spectroscopy
GO	Graphen Oxide
DLS	Dynamic light scattering
IR	Infrared radiation
CA	Citric acid
DDS	Sodium dodecyl sulfate
PSS	Sodium polystyrene sulfonate
CS	Chitosan
CTAB	Cetyltrimethylammonium bromide
