# Supplementary material for: Photothermal Evaluation of Aqueous Magnetite Nanodispersions: Accuracy, Precision, and Limitations
Source: Molecules. 2025 Oct 14;30(20):4084. doi: 10.3390/molecules30204084 (PMC12565839; doi:10.3390/molecules30204084)
Supplement: Supplementary file 1 [file molecules-30-04084-s001.zip › molecules-3855359-supplementary.pdf]

molecules

MDPI

Supplementary materials

Photothermal evaluation of aqueous magnetite nanodispersions: accuracy, precision, and limitations

Vladislav R. Khabibullin [1], Daria-Maria V. Ratova [1], Ksenia O. Andreeva [2], Yulia S. Vershinina [1,3], Ivan V. Mikheev [1*], Sergei N. Shtykov [2] and Mikhail A. Proskurnin [1*]

[1] Analytical Chemistry Division, Chemistry Department, M. V. Lomonosov Moscow State University, d. 1, Str. 3, Lenin Hills, GSP-1, Moscow 119234, Russia; vladhab1995@gmail.com (V.R.K.); darmarrat@gmail.com (D.-M.V.R.); mikheev.ivan@gmail.com (I.V.M.); proskurnin@gmail.com (M.A.P.);

[2] Department of Analytical Chemistry and Chemical Ecology, Institute of Chemistry, Saratov State University, Saratov 410012, Russia; kazimirova-ks@mail.ru (K.O.K); shtykovsn@mail.ru (S.N.S);

[3] Federal State Budgetary Institution of Science Institute of African Studies, Russian Academy of Sciences, Spiridonovka St., 30/1, 123001 Moscow, Russia; yu.vrshn@gmail.com (Y.S.V.);

* Correspondence: mikheev.ivan@gmail.com (I.V.M.); proskurnin@gmail.com (M.A.P.);

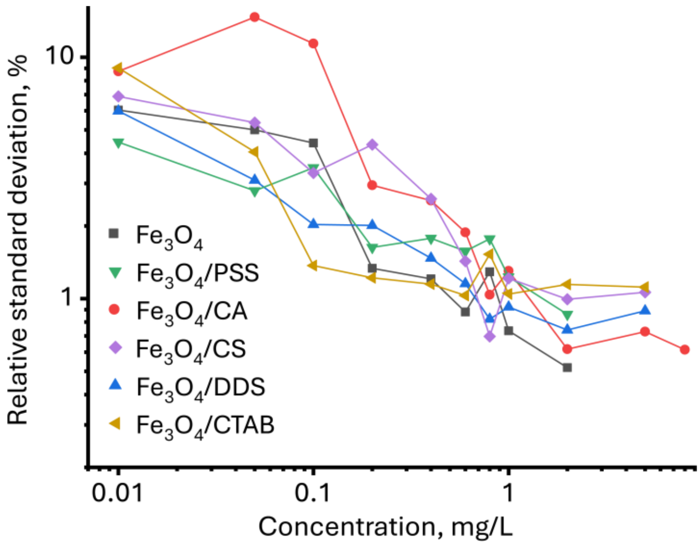

Figure S1. Relative standard deviation of thermal diffusivity measurements of magnetite nanoparticle dispersions ($n = 3$).

Synthesis of magnetite nanoparticles

Magnetite nanoparticles (Fe_3O_4) were obtained by chemical co-precipitation. First, an alkaline solution was prepared by dissolving 2.0 g of NaOH in 150 mL of deionized water under nitrogen atmosphere. Then, a solution of iron (II) and (III) salts was made up by dissolving 1.30 g of $FeCl_3 \times 6H_2O$ and 0.48 g of $FeCl_2 \times 4H_2O$ in 35 mL of deionized water under nitrogen atmosphere. This solution was added to the NaOH solution heated to 40◦C and constantly stirred at 1800 rpm. Next, a nitrogen atmosphere was passed through the mixed solution for 30 min, as a result the colloidal solution changed color from light ye to black.

Surface modification of magnetite nanoparticles by CTAB, DDS, and PSS

The resulting Fe_3O_4 washed several times with deionized water and ethanol, and dispersed in 12.5 mL of a solution of CTAB, DDS or PSS with a concentration of 0.06 M under the action of ultrasound for 30 min

Surface modification of magnetite nanoparticles by chitosan

60 mL of chitosan (CS) with a concentration of 2% (w/w) in an acetic acid solution were added to the magnetic magnetite nanoparticles and stirred for 30 min at a speed of 1800 rpm under nitrogen at a temperature of 60°C. The resulting black precipitate of modified magnetite nanoparticles was precipitated using a permanent magnet and the supernatant was decanted.

Surface modification of magnetite nanoparticles by CA

Initially, a solution of the reagents used was prepared: 1.30 g $FeCl_3{\cdot}6H_2O$ and 0.48 g $FeCl_2{\cdot}4H_2O$ were dissolved at room temperature in 12 ml of water with stirring, 2 g NaOH was dissolved at room temperature in 50 ml of water. The resulting solutions were subjected to ultrasonication for 10 minutes at 25°C. 20 mL of 1 M NaOH and 150 ml of water were placed in the reaction chamber. The temperature (40°C) and rotation speed (1800 rpm) were set. Nitrogen was bubbled through the reaction chamber for 15 minutes to remove dissolved oxygen. Then, a solution of iron salts was injected under nitrogen pressure into the actively stirred sodium hydroxide solution. 3 minutes after the introduction of the salts, 20 mL of citric acid (40 mg/ml) were added and stirring of the solution was continued for 2 minutes

X-ray Diffraction

Figure S2 shows the XRD spectra of Fe_3O_4 and chitosan, SDS and PSS coated magnetite. The presence of six characteristic peaks of Fe_3O_4 at 2θ ~ 30.1, 35.5, 43.1, 53.4, 57.0 and 62.6 units in the XRD pattern, marked with indices (220), (311), (400), (422), (511), respectively, were observed for all the samples. The results are in good agreement with the standard crystal XRD data card (JCPDS No. 85-1436), which confirms that the obtained magnetite NPs correspond to the spinel structure. The results indicate that the Fe_3O_4 core did not change the crystal structure after the functionalization of the nanoparticles. In addition, the modification process did not lead to a phase change for the Fe_3O_4 core.

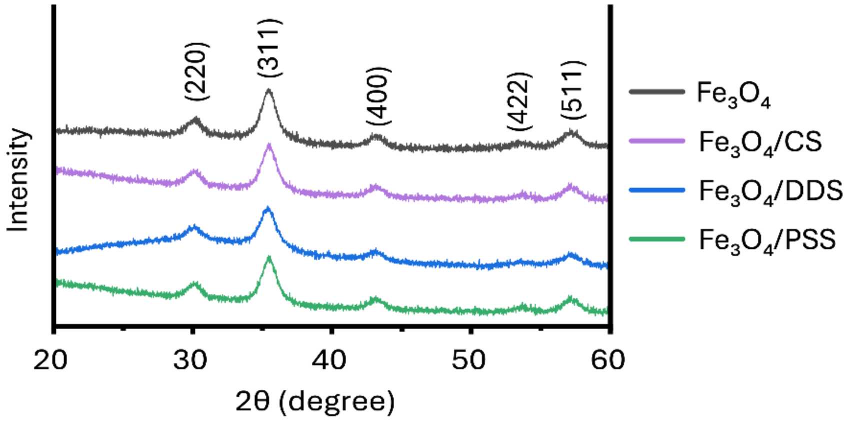

Figure S2. Diffraction pattern of uncoated and chitosan, PSS and DDS-modified magnetite nanoparticles.

The Debye–Scherrer equation was used to estimate the crystallite size of magnetite nanoparticles:

$$D = \frac{k\lambda}{\beta \mathrm{Cos}(\theta)}, \quad (1)$$

where k is the shape factor, λ is the X-ray wavelength, β is the full width at half maximum intensity, and θ is the Bragg angle.

Table S1 presents the results of the analysis and crystallite size estimation using the Williamson–Hall method.

Table S1. Estimation of crystallite sizes by the Williamson-Hall method.

Sample	Crystallite size, nm
Fe_3O_4	5.1
Fe_3O_4/DDS	6.2
Fe_3O_4/CS	7.7
Fe_3O_4/CTAB	8.5

Transmission electron microscopy

The synthesized materials are a polydisperse ensemble of magnetite particles. The particle size for unmodified magnetite Fe_3O_4, determined from TEM images, is in the range of 5–10 nm. The calculated d values were limited to a sample of 500 particles. Thus, the average particle size is 7.5 ± 2.5 nm (Figure S3). Larger aggregates are also visible in the TEM micrographs (>30 nm).

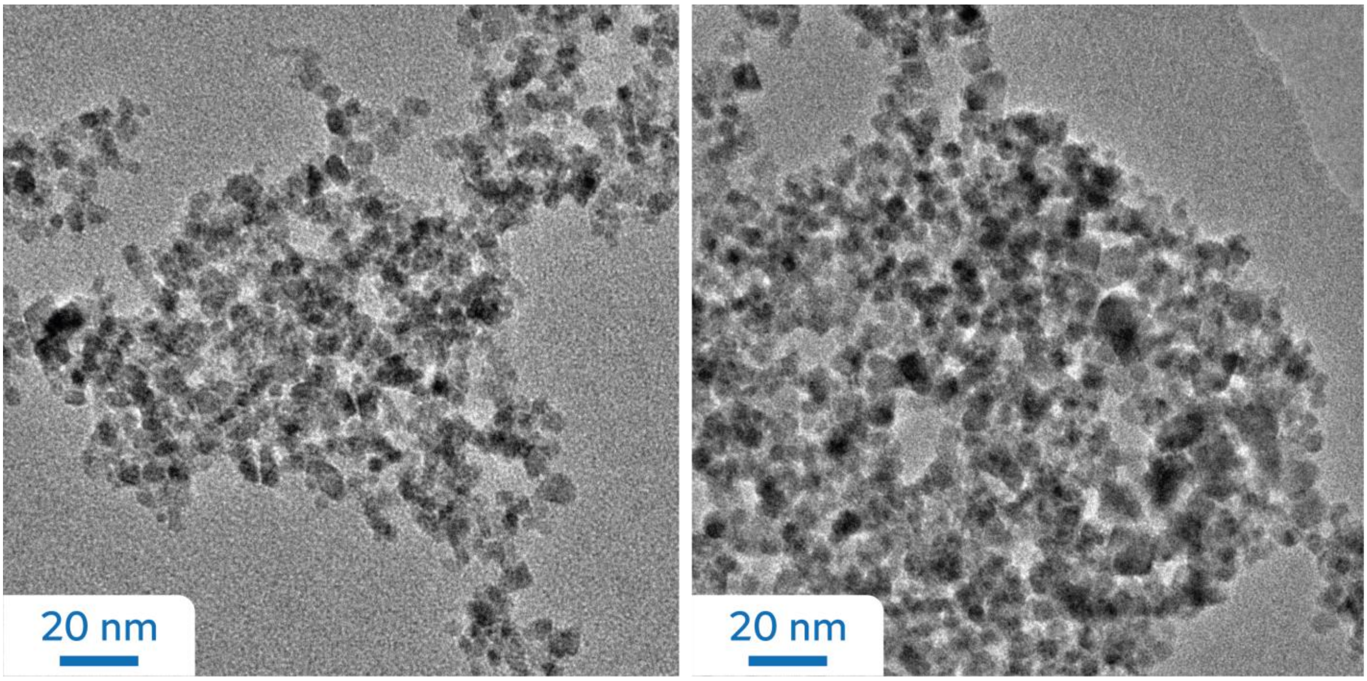

Figure S3. TEM images for the Fe_3O_4.

Zeta Potential and Hydrodynamic Size Measurement

The results of measuring the Zeta-potential (ζ) and average hydrodynamic size (*Z*-average) of the functionalized magnetite NPs in deionized water are presented in Table S2. For particles modified with citric acid, chitosan and polystyrene sulfonate, ζ exceeds the accepted stability threshold ($\zeta > |30|$ mV [1]) and stable behavior in suspensions can be expected. Nanoparticles coated with citric acid, SDS and PSS have negative ζ, which is associated with the presence of carboxyl and sulfo-groups on the surface. Nanoparticles coated with chitosan and CTAB, on the contrary, have positive ζ, close to the stability threshold, due to the amino groups.

Table S2. Average hydrodynamic size and zeta potential of magnetite nanoparticles with different surface modifiers.

Sample	ζ[1], mV	*Z*-average size, nm	PDI
Fe_3O_4	9.6 ± 0.7	107	0.223
Fe_3O_4/CA	–38 ± 2	353	0.413
Fe_3O_4/DDS	–23 ± 2	4260	0.261
Fe_3O_4/PSS	–41 ± 3	3054	0.321
Fe_3O_4/CS	35 ± 3	4270	0.477
Fe_3O_4/CTAB	29 ± 2	5025	0.315

[1] Average of ζ values from three measurements.

A significant difference in the *Z*-average size between the particles of coated and uncoated magnetite indicates successful surface functionalization. At the same time, a low value of the polydispersity index PDI indicates a monodisperse nature of the particles obtained after modification.

Infrared Spectroscopy

The spectrum of Fe_3O_4 NPs (Fig. S4 A) exhibits the following features: a broad νOH stretching band between 3500 and 3100 cm^{-1} and β_{OH} (in H_2O) bending mode at 1630 cm^{-1}, both arising from adsorbed water. The series of bands in the 2700–2300 cm^{-1} region may be due to O–H stretching vibrations in COOH dimers CO_2 absorption bands. A band at 1530 cm^{-1} is assigned to antisymmetric stretching vibrations of COO^- groups, and the

band at 1335 cm⁻¹ to the corresponding symmetric stretching vibrations [2]. Intense bands at 620 and 535 cm⁻¹ correspond to the Fe–O lattice vibrations of the nanoparticles [3].

The spectra of the doped nanoparticles show clear evidence of binding with CA, CS, CTAB, and PSS (Figure S4 B-D and F). In contrast, no new bands appear in the spectrum of the DDS-treated sample (Figure S4 E), indicating that DDS does not bind to the nanoparticle surface. Notably, in the CA-coated nanoparticles (Figure S4 B), the bands at 1590 and 1350 cm⁻¹ are both shifted and significantly intensified, consistent with strong ligand–surface coordination.

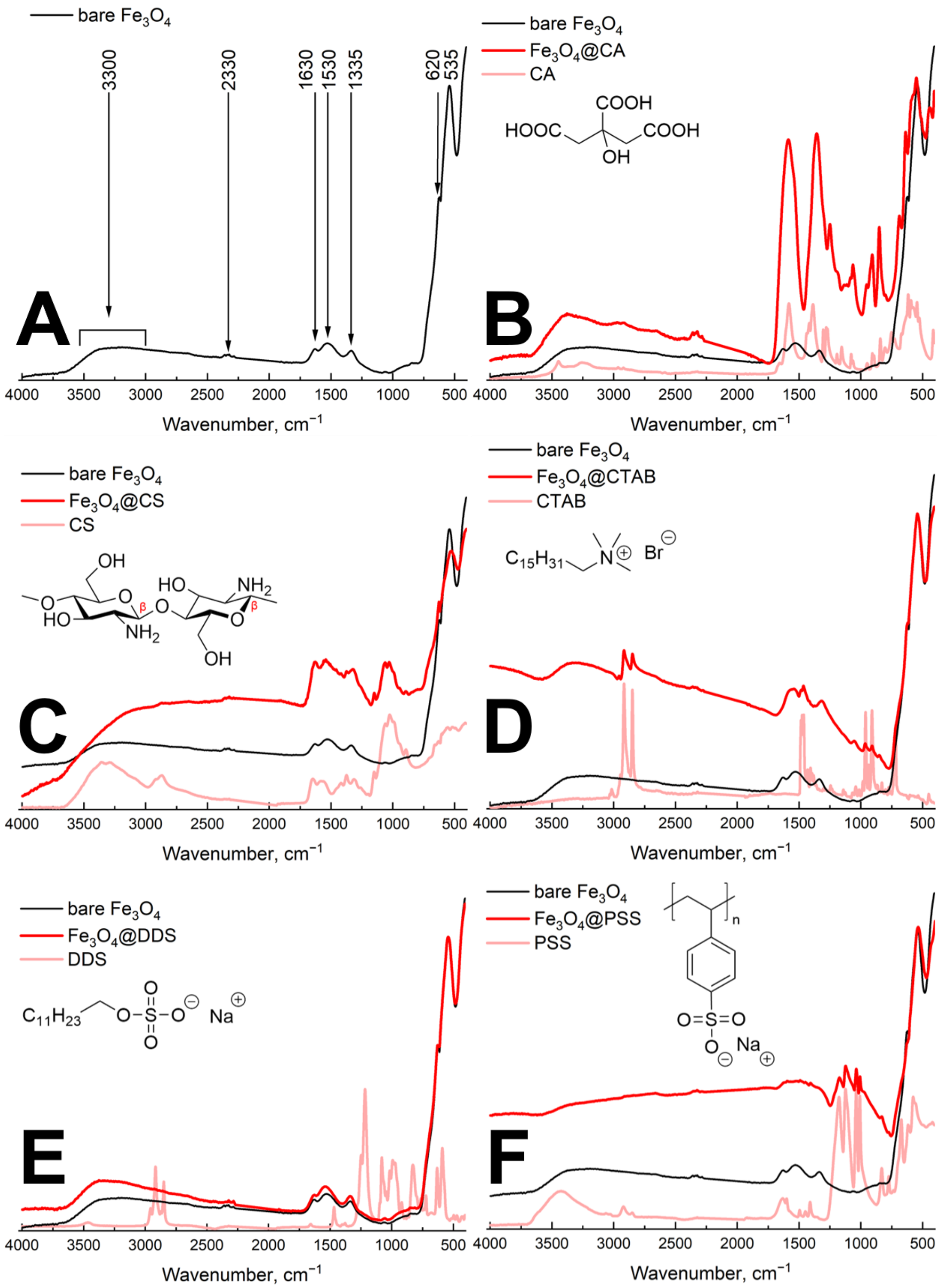

Figure S4. ATR-FTIR spectra in the absorption mode from 4000 to 400 cm⁻¹ (64 scans, 2 cm⁻¹) of: (**A**) pure nanoparticles; (**B**) particles doped with CA; (**C**) particles doped with CS; (**D**) particles doped with CTAB;(**E**) particles doped with DDS; (**F**) particles doped with PSS

Photothermal measurements

Table S3. Thermal-lens measurement parameters

Parameter	Value
Excitation laser	
Wavelength, λ_e (nm)	532
Focussing lens focal length, f_e (mm)	200
Confocal distance, Z_{ce} (mm)	10.4
Laser power at cell, P (mW)	200-300
Spot size at the waist, ω_{e0} (μm)	42
Probe laser	
Wavelength λ_p (nm)	632.8
Focusing lens focal length f_p (mm)	300
Confocal distance, Z_{cp} (mm)	2.7
Laser power at cell (mW)	6.0
Spot size at the waist, ω_{p0} (μm)	23
Spot size at cell, ω_p (μm)	510
Other constants	
Cell length (mm)	10
Sample-to-detector distance, Z_2 (cm)	230
Mode mismatch factor m	147
Geometric parameters V'	36.2
Modulator frequency (Hz)	1
Number of transient curves to average	300

References

1. Schramm, L.L. *Emulsions, Foams, Suspensions, and Aerosols: Microscience and Applications*; Wiley: 2014.
2. Yıldırım, E.; Arıkan, B.; Yücel, O.; Çakır, O.; Kara, N.T.; İyim, T.B.; Gürdağ, G.; Emik, S. Synthesis and characterization of amino functional poly(acrylamide) coated Fe3O4 nanoparticles and investigation of their potential usage in DNA isolation. *Chemical Papers* **2022**, *76*, 5747-5759, doi:10.1007/s11696-022-02293-y.
3. Singh, S.; Goswami, N. Structural, optical, magnetic and dielectric properties of magnetite (Fe3O4) nanoparticles prepared by exploding wire technique. *Journal of Materials Science: Materials in Electronics* **2021**, *32*, 26857-26870, doi:10.1007/s10854-021-07062-3.
